# When protein losing enteropathy persists: A case series of viral and lymphatic‐associated etiologies

**DOI:** 10.1002/jpr3.70150

**Published:** 2026-02-05

**Authors:** Natalie Jennings, Antonia C. Rovira, Pamela Castillo Rocha, Janelle Torres, Claudia M. Salgado, Miguel Reyes‐Múgica, Richard N. Arboleda, Fatima S. Hussain

**Affiliations:** ^1^ Nicklaus Children's Hospital Miami Florida USA; ^2^ Division of Pediatric Gastroenterology, Hepatology and Nutrition University of Miami Miami Florida USA; ^3^ Department of Pathology and Laboratory Medicine University of Miami Miami Florida USA; ^4^ Pediatric Gastroenterology Associates Nicklaus Children's Hospital Miami Florida USA; ^5^ Department of Pediatrics, Division of Pediatric Gastroenterology University of Saskatchewan Saskatoon Saskatchewan Canada

**Keywords:** alpha‐1‐antitrypsin, diarrhea, primary intestinal lymphangiectasia

## Abstract

Protein‐losing enteropathy (PLE) is a rare condition that is characterized by loss of plasma protein in the intestines leading to hypoproteinemia with subsequent peripheral edema and possibly anasarca. The pathophysiology of PLE varies depending on the etiology and involves either intestinal mucosal injury or lymphatic system alterations. While transient PLE can occur in the setting of viral infections, persistent or recurrent PLE requires comprehensive evaluation and multidisciplinary management approach. Here, we present two cases of PLE: the first case secondary to a common pediatric infection and the second case of primary intestinal lymphangiectasia with severe symptoms improved with diet therapy.

## Abstract (highlights)

**What is Known:**

- Protein‐losing enteropathy (PLE) is characterized by loss of serum proteins in the gastrointestinal tract causing hypoproteinemia with subsequent edema.
- The diagnosis of protein losing enteropathy is confirmed with elevated stool alpha‐1‐antitrypsin.

**What is New:**

- PLE secondary to an infectious etiology that resolves with supportive care typically does not require further diagnostic evaluation.
- Viral associated protein losing enteropathy may be considered in a child with a short clinical history.

## 1 INTRODUCTION

Protein‐losing enteropathy (PLE) is a disease characterized by excessive loss of serum protein into the gastrointestinal (GI) tract that can result in hypoproteinemia and, subsequently, edema, nutritional deficiencies, and growth failure in pediatric patients. The pathophysiology of PLE includes alterations to the GI mucosa in the setting of inflammation or injury, malabsorption, increased mucosal or vascular permeability, or lymphatic abnormalities.^1^ In children, PLE has been diagnosed following infections with severe acute respiratory syndrome coronavirus 2 (SARS‐CoV‐2), cytomegalovirus, *Clostridium difficile*, human immunodeficiency virus (HIV), and rotavirus infection.^2^, ^3^, ^4^, ^5^ In addition, PLE secondary to norovirus has been reported in immunocompromised patients.^5^ Influenza virus has not been reported as an etiology of PLE, however it can cause intestinal inflammation as with other viruses, and thus disrupting the intestinal barrier.^6^ On the other hand, lymphatic abnormalities represent a major category of PLE etiology. These may be primary (congenital) or secondary, due to lymphatic obstruction, elevated lymphatic pressure, or as part of certain syndromic presentations.^1^ Secondary lymphangiectasia has also been documented as a rare but life‐threatening complication of palliative surgeries for congenital heart disease. Primary intestinal lymphangiectasia (PIL), otherwise known as Waldmann's disease, as a cause of PLE is rarely described in the literature. It is characterized by hypoalbuminemia with subsequent edema due to dilation of lymphatic vessels in the intestinal mucosa and submucosa.^1^, ^7^ The purpose of this case series is to highlight two otherwise healthy patients who developed PLE, 1 secondary to norovirus and the other initially thought to be due to viral causes, but after presenting with recurrent PLE episodes, was diagnosed with PIL.

## 2 METHODS

We report two cases of Protein Losing Enteropathy managed at Nicklaus Children's Hospital and/or University of Miami between 2023 and 2024. Both cases had PLE confirmed by elevated stool alpha‐1‐antitrypsin. Institutional Review Board approval was waived.

### 2.1 Ethics statement

Verbal informed consent was obtained by the parents of the patients (as both patients are minors) that this case will appear/be published in a journal. Discussed the content details with them, and they expressed understanding and consented for the case report to be published.

## 3 RESULTS

### 3.1 Case 1

A 12‐month‐old full‐term female with no past medical history presented with a 9‐day history of nausea, vomiting, loose stools, edema of the bilateral feet and eyelids, and refusal to crawl or walk. Two days into her disease course, she began having mild swelling of the feet that had progressively worsened. On admission, her complete blood count was within normal limits, Albumin 1.4 gm/dL, with alanine aminotransferase (ALT) and aspartate transaminase (AST) 52 and 68 IU/L, respectively. Immunoglobulin (Ig) levels were not collected. Her GI stool pathogen panel was positive for *C. difficile* toxin A/B, enterotoxigenic *Escherichia coli* (ETEC), and norovirus. *C. difficile* glutamate dehydrogenase antigen was not obtained as that test was not available at our institution at the time. Alpha‐1‐antitrypsin in the stool was 295 mg/dL (reference range <55 mg/dL), supporting the diagnosis of PLE. Renal, hepatic, cardiac, and malnutrition causes of the edema were ruled out during hospitalization. Throughout admission, her diarrhea and edema improved with supportive measures, therefore treatment of her identified stool pathogens was deferred. Esophagogastroduodenoscopy (EGD) with flexible sigmoidoscopy was not performed. The patient returned for follow‐up 2 weeks later, reporting occasional loose stools but no edema, which coincided with other viral symptoms at the time. Repeat testing showed an albumin level of 3.8 g/dL and an AST level of 38 U/L. Although stool alpha‐1‐antitrypsin testing was ordered, it was never completed, and the patient subsequently did not return for further follow‐up.

### 3.2 Case 2

A 16‐month‐old, full‐term female with no significant past medical history presented with a 1‐week history of persistent cough and periorbital edema that then developed into anasarca. One month prior to presentation, she had an upper respiratory infection secondary to influenza A virus. Laboratory results were significant for albumin of 1.4 g/dL, decreased Ig (IgE, IgM, and IgG), and stool alpha‐1‐antitrypsin of 1880 mg/dL. Stool occult blood was initially positive; however, repeat testing was negative. She had an elevated stool calprotectin of 660 mcg/g. Infectious workup was otherwise negative and Celiac disease serology was negative. Cardiology, nephrology, endocrinology, and hepatology evaluation were all unremarkable. She received an intravenous Ig infusion for hypogammaglobulinemia and albumin infusion for hypoalbuminemia. EGD with flexible sigmoidoscopy were performed and revealed lymphoid hyperplasia in the duodenum and sigmoid colon which were not indicative of inflammatory bowel disease, but otherwise thought to be non‐specific, supporting the diagnosis of PLE secondary to infectious cause. The patient was treated with supportive measures. Her symptoms improved during her hospital admission and the patient was discharged home.

**Figure 1:**
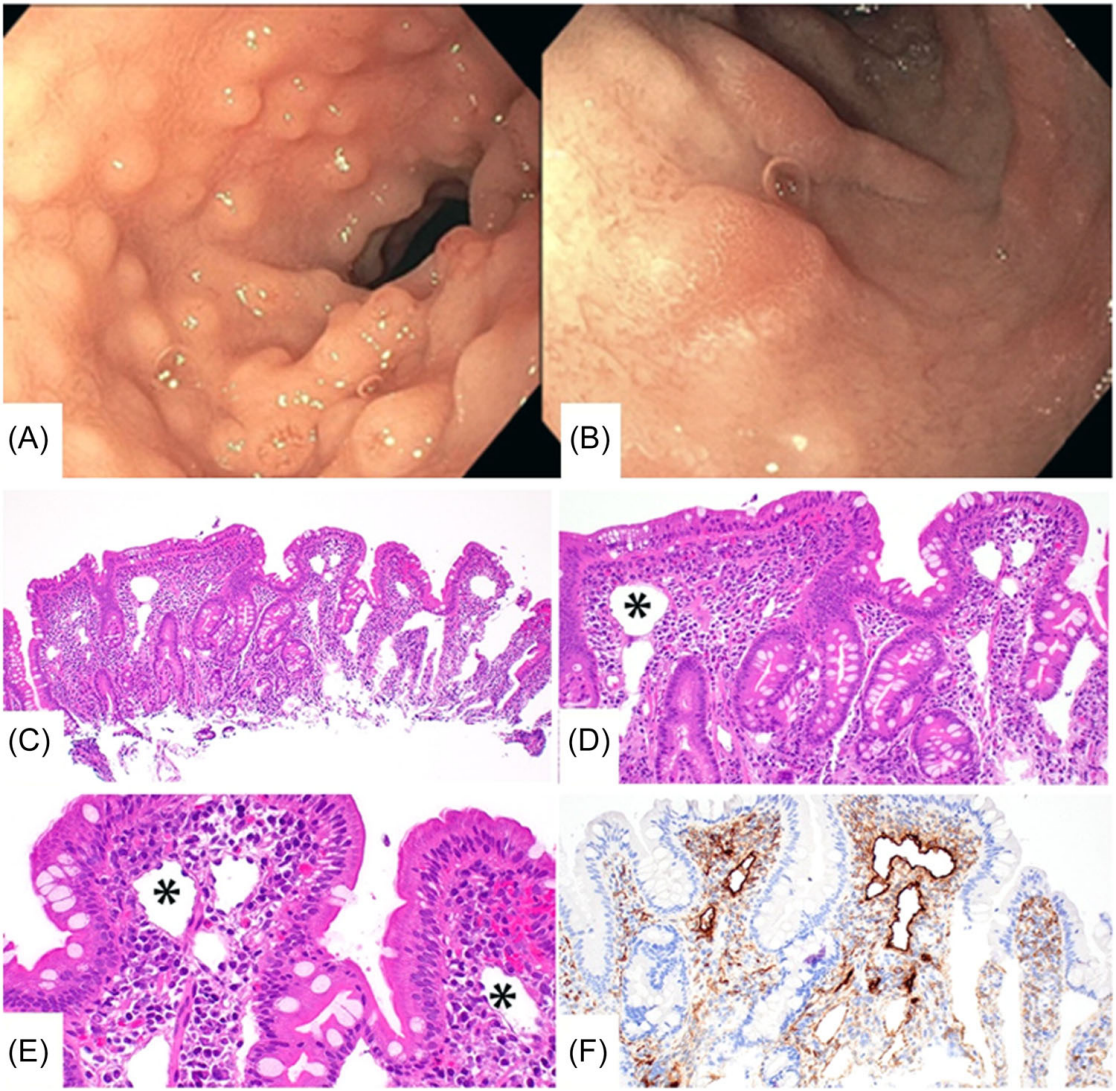
(A) Duodenal bulb on EGD showing nodular mucosa. (B) Second portion of the duodenum on EGD showing plaques of edematous white tipped villi. (C–E) Photomicrographs of the duodenal mucosa with dilated lymphatics labeled with asterisks (original magnifications C: ×10; D: ×20; E: ×40). (F) The lymphatic vessels endothelium highlighted in brown with an immunohistochemical stain for D2–40 (original magnification ×20). EGD, esophagogastroduodenoscopy.

The patient then presented two additional times for severe anasarca with hypoalbuminemia, both in the setting of acute upper respiratory infections, first secondary to rhinovirus/enterovirus 1 month later, then coronavirus HKU1 the following month. Her alpha‐1‐antitrypsin remained elevated up to 985 mg/dL with severe hypoalbuminemia down to 1.4 g/dL. Given her recurrent symptoms, her workup was expanded to evaluate for a genetic or oncologic cause. Computed tomography of the abdomen was negative for tumors causing lymphatic obstruction. Genetic workup showed a pathogenic variant in PLE associated with autosomal dominant angioedema, which was deemed not a causative factor of the anasarca and clinical presentation. Finally, the patient underwent a repeat EGD and colonoscopy 6 months after her initial presentation which macroscopically showed nodular mucosa in the duodenum with plaques of edematous tissue with white‐tipped villi (Figure 1A,B), and pathology of the biopsied areas showed duodenal mucosa with mildly dilated lymphatic vessels highlighted by immunohistochemistry for D2–40, with no significant villous atrophy or signs of chronic inflammation (Figure 1C–F). These findings were consistent with a diagnosis of PIL. The patient was started on high‐protein and low‐fat diet with medium‐chain triglycerides (MCT) guided by a GI dietician with a goal of 24 g of protein per day. Her treatment plan also initially included a trial of octreotide as a second‐line therapy; however, the patient improved significantly on dietary therapy and did not start the octreotide or any further pharmacologic treatment. She has had no further episodes of edema or hypoalbuminemia and was growing well at her most recent outpatient follow‐up appointment 1 year after diagnosis.

## 4 DISCUSSION

Inflammation of the intestinal barrier due to infection is a known cause of PLE, and many different viruses have been implicated, including norovirus, SARS‐CoV‐2, and rotavirus. However, if a patient is presenting with repeated episodes of edema, other etiologies should be considered as the underlying cause of the PLE for treatment optimization. In our case series, the patients had similar initial presentation with non‐specific upper respiratory or GI symptoms followed by generalized edema and hypoalbuminemia. Case one's symptoms resolved with supportive care and thus were likely secondary to her multiple GI infections, although the positive C. difficile test was likely due to carrier status given her age, however could not be confirmed via antigen testing. However, while the second case also had multiple viral infections that were initially implicated in the etiology of her PLE, presentation with edema and hypoalbuminemia continued to recur, highlighting the importance of further workup to determine an alternative etiology. On her initial workup, the endoscopy only showed non‐specific findings of lymphoid hyperplasia in the duodenum and sigmoid colon, which did not elucidate an underlying etiology of her PLE at that time. In the setting of a likely infectious etiology, it was thought to be non‐contributory. It was only after the recurrence of symptoms that genetic and immunologic workup was initiated, and endoscopy was repeated, identifying an unrelated but important pathogenic variant in PLE and finally the underlying etiology of her PLE; PIL.

PIL is characterized by defects in the intestinal lymphatic system not otherwise caused by cardiac, inflammatory, or oncologic disease. These abnormalities may cause obstruction of lymphatic vessels, leading to increased intestinal lymphatic pressure. This elevated pressure can cause lymphatic leakage in the intestines, resulting in protein losing enteropathy.^7^, ^8^ PIL is diagnosed via endoscopy, which reveals dilated lacteal vessels that distort villous architecture, and confirmed through histological examination showing both lacteal dilation and villous blunting.^8^ Treatment of PLE is directed by its underlying etiology. For patients with viral etiologies, such as our patient in case one with norovirus, treatment is largely supportive. This includes hydration, albumin infusions, and even diuretics to manage edema. On the other hand, the underlying pathophysiology for PIL is unknown with few genetic variants identified in the literature. There is no treatment pathway or guidelines for the treatment of PIL but several treatment modalities have been used depending on the severity of symptoms and tolerance of the patients. Suggested treatment options include corticosteroids,^9^ immunosuppressants (e.g., Everolimus) or Octreotide, as well as dietary management with a high‐protein, low‐fat diet supplemented with MCTs. This diet works by reducing lymphatic flow and thus protein loss. Salvia et al. described a case in which a preterm infant was diagnosed with intestinal lymphangiectasia which subsequently resolved with the replacement of formula with one with a high concentration of medium chain triglycerides.^8^ If there is a component of malabsorption, supplementation with fat‐soluble vitamins and micronutrients may be necessary. Interestingly, Ozeki et al. describes a patient with PLE refractory to dietary changes and propranolol but had improvement with the initiation of Everolimus.^9^ Kwon et al. reported the use of octreotide in several patients who responded either to initial induction period or maintenance over 1 year with recurrence of symptoms afterwards.^10^ Although both of our cases did have recurrent viruses on presentation, it is important to investigate other causes of PLE when there is no improvement with supportive care, or if no viral source is detected.

## 5 CONCLUSION

Protein losing enteropathy can be caused by a multitude of etiologies, leading to either inflammation and subsequent disruption of the intestinal barrier or malfunction of the lymphatic system. Correct diagnosis of the underlying etiology is paramount for appropriate treatment. While a single episode that resolves with supportive treatment in the context of a viral infection may not warrant further investigation, recurrent episodes, episodes without a clear viral source, or those unresponsive to supportive treatment should prompt a more thorough evaluation to rule out underlying or contributing causes. Although rare, PIL should be considered in these cases, with prompt diagnosis leading to targeted therapy, faster recovery, and a better prognosis.

## CONFLICT OF INTEREST STATEMENT

The authors declare no conflicts of interest.
